# A combination of the HLA-DRB1*03 phenotype and low plasma mannose-binding lectin predisposes to autoantibody formation in women with recurrent pregnancy loss

**DOI:** 10.3389/fimmu.2023.1069974

**Published:** 2023-01-26

**Authors:** Caroline Nørgaard-Pedersen, Rudi Steffensen, Ulrik Schiøler Kesmodel, Ole Bjarne Christiansen

**Affiliations:** ^1^ Centre for Recurrent Pregnancy loss of Western Denmark, Department of Obstetrics and Gynaecology, Aalborg University Hospital, Aalborg, Denmark; ^2^ Department of Clinical Medicine, Aalborg University, Aalborg, Denmark; ^3^ Department of Clinical Immunology, Aalborg University Hospital, Aalborg, Denmark

**Keywords:** HLA class II, mannose-binding lectin, autoantibodies, antiphospholipid antibodies, recurrent pregnancy loss, habitual abortion

## Abstract

**Introduction:**

It is documented that a series of autoantibodies can be detected with increased frequency in women with recurrent pregnancy loss (RPL) and they may impact the pregnancy prognosis negatively. It is unknown whether the autoantibodies per se or the basic immune disturbances underlying autoantibody production, are the reason for this association. Our group has previously found that some genetically determined immunological biomarkers are associated with RPL and the same biomarkers are also in various degrees known to predispose to autoantibody production. The aim of this study was to clarify whether the RPL-associated immunogenetic biomarkers are associated with positivity for three major classes of autoantibodies associated with RPL.

**Methods:**

In 663 patients with RPL in whom we had results for HLA-DRB1 typing and plasma mannose-binding lectin (p-MBL) measurement, it was investigated whether there is a correlation between positivity for the autoantibodies: anticardiolipin antibodies, β2 glycoprotein I antibodies, and lupus anticoagulant (jointly called antiphospholipid antibodies), thyroid-peroxidase antibodies, and antinuclear antibodies and each of the HLA-DRB1 alleles HLA-DRB1*03 or HLA-DRB1*07 either alone or in combination with low p-MBL defined as ≤500 µg/l.

**Results:**

Although slightly higher frequencies of positivity of two or more autoantibodies were seen in patients with either p-MBL ≤500 µg/l or being positive for HLA-DRB1*03, none were significantly associated. However, in patients with the combination of low p-MBL and HLA-DRB1*03, presence of at least one autoantibody was significantly more frequent than in patients with no such combination (OR= 2.4; 95% CI 1.2-5.0, p = 0.01). In an analysis of which autoantibodies were most strongly associated with the low p-MBL/HLA-DRB1*03 combination, antinuclear antibodies were significantly more frequent in these patients (OR 2.0; 95% CI 1.0-3.9, p=0.05) whereas the other autoantibodies were also positively but more weakly associated with this combination.

**Discussion:**

In conclusion, to clarify the pathogenetic background, underlying immunogenetic factors should be examined in autoantibody positive RPL patients (as well as other patients with autoimmune diseases) but the genetic background may be complex.

## 1 Introduction

Autoimmune diseases are manifestations of immune system dysregulation caused by a humoral or cell-mediated immune response against self-antigens and consequently loss of self-tolerance. This heterogenous group of diseases shares some common pathophysiological mechanisms and risk factors which involve genetic, environmental, immunological, and hormonal factors that contribute to the tissue injury and clinical manifestations (1). Among the most relevant and studied genetic components in autoimmune diseases are the loci coding for human leucocyte antigens (HLA) class I and II, which are located on the short arm of chromosome no. 6. In particular, HLA class II alleles - and more specifically HLA-DRB1 alleles - are considered to be more strongly involved in the susceptibility to or protection against specific autoimmune diseases than HLA class I (2, 3). However, strong associations between HLA alleles and autoantibody positivity also exist, and antibody positivity often precedes clinical manifestation of autoimmune disease by several years or decades. Therefore, one may question whether the primary role of HLA genes is predisposition to the disease through non-humoral pathways or antibody development that secondarily leads to autoimmune diseases (4).

One example of an association between HLA alleles and autoantibody positivity, is anti-citrullinated protein antibodies in patients with rheumatoid arthritis. These antibodies are mainly found in patients with a specific 5 amino acid motif located in the β-chain of the HLA-DRB1 alleles that predisposes to rheumatoid arthritis (5). Other examples are that specific HLA-DQ alleles are associated with disease-specific transglutaminase 2 antibodies in celiac disease (6); HLA-DRB1*0401 is strongly associated with autoantibodies against insulin and islet antigen 2 proteins (7); while the HLA class II haplotype HLA-DRB1*03-DQB1*02 is associated with glutamic acid decarboxylase antibodies in type 1 diabetes mellitus (8). However, the genetic background for development of autoimmune disease and autoantibody production is complex. In addition to the HLA genes, non-HLA related genetic factors and environmental factors play a role (2).

One of the non-HLA genetically determined biomarkers of autoimmunity is mannose-binding lectin (MBL) deficiency, which is mainly caused by the interaction of several gene polymorphisms on chromosome no. 10. Plasma (p-)MBL deficiency has been associated with the development and severity of several autoimmune diseases such as lupus erythematosus and rheumatoid arthritis (9–11).

In accordance with the Recurrent Pregnancy Loss (RPL) guideline of the European Society of Hunan Reproduction and Embryology (12), RPL is in this study defined as 2 or more consecutive pregnancy losses but the definition of RPL varies between different RPL guidelines and specialist societies (12, 13). In about 50% of patients, thrombophilia or endocrine, anatomic, or genetic risk factors can be found, while in the remaining subgroup of patients an aberrant immune profile is often found (14, 15). This involves autoantibody positivity or disrupted leucocyte subset distribution or function. Anti-nuclear antibodies (ANA), anti-thyroid peroxidase (TPO) antibodies and anti-phospholipid (APL) antibodies are associated with significantly increased odds ratios (OR) for RPL (16–20). Maternal carriage of HLA-DRB1*03 (21) and HLA-DRB1*07 (22) alleles and p-MBL deficiency (23, 24) have also been reported to be associated with RPL.

Due to the well-known association between HLA class II alleles and autoantibody production, as well as the association between p-MBL deficiency and some autoimmune diseases, we found it important to investigate whether autoantibody formation is an intermediate variable between the two RPL-associated genetic factors HLA-DRB1 and low p-MBL and RPL.

## 2 Materials and method

### 2.1 Population

Patients with RPL consecutively referred to the Center for Recurrent Pregnancy Losses in Western Denmark (in the following called the RPL Center) from January 2016 to August 2022 were included. RPL was defined as two or more consecutive pregnancy losses. Patients with known chromosomal abnormalities (N=11), uterine malformations (N=12), or missing analysis for autoantibody or HLA-DRB1 (N=22) were excluded. All patients were diagnosed at the RPL Center and all participants had given an oral and written consent to the investigators for storing their data in an RPL database. During the diagnostic work-up, information on the general health, gynaecologic and obstetric history, and lifestyle was collected as well as a routine blood sample analyzed for several factors including p-MBL level, HLA-DRB1 genotype, and presence of APL antibodies (lupus anticoagulant (LA), anti-cardiolipin antibody (aCL), β2-glycoprotein-I (β2GPI) antibody), ANA, and anti-TPO. Patients were diagnosed as APL syndrome positive if they were positive for LA or had aCL or β2GPI antibody (levels ≥35 kU/I) detected twice three to four weeks apart. The patients’ ethnic origin was not noted in the database but the prevalence of non-Caucasians was estimated by the clinicians to be <2%. As patients referred to the RPL clinic are recommended not to get pregnant when waiting for their diagnostic work-up, only 10.2% of patients were pregnant in the first trimester at the time the blood sample was collected.

### 2.2 Ethical issue

Data was extracted from the RPL database (North Region Approval Number: 2018-5). Since only data on routine investigations and interventions in the RPL Center were analysed and reported, no permission from the ethics committee was required.

### 2.3 Blood sample analysis

The p-MBL level was determined with the enzyme-linked immunosorbent assay (ELISA) method as described by Nørgaard-Pedersen et al. (23). A low p-MBL level was defined as ≤500 µg/l, which is the cut-off value used routinely in Danish laboratories.

HLA-DRB1 typing was performed by the FluoGene system combining polymerase chain reaction (PCR) using sequence-specific primer (PCR-SSP) with the speed of endpoint fluorescence detection. The analysis was based on specifically modified TaqMan probe system (Iinno-train Diagnostik GmbH). The PCR was performed according to the manufacturer’s instructions and the subsequent pre- and postreads are generated on a FluoVista and finally was the HLA-DRB1 type concluded by the FluoGene Software.

Indirect immunofluorescence assay on HEp-2 cells was used to detect numerous of autoantibodies against cytoplasmatic or nuclear antigens, among which are i.e., autoantibodies against double-stranded DNA (ds-DNA), SS-A/Ro, SS-B/La, Sm, Scl-70, Jo-1, U1RNP (RNP70, A, C), Ribosomal P, PCNA, fibrillarin, RNA polymerase III, PM-Scl100, Mi-2, centromere B, etc. The AESKUSLIDES^®^ ANA-HEp-2 instruction manual was followed, and the laboratory used a screening titer of 1:160 and the 51.100 ANA HEp-2 standard kit. The samples were screened for ANAs using the Helios reader (AESKU, Mikroforum Ring 2, 55234 Wendelsheim, Germany), a fully automated Indirect immunofluorescence processor, and when one in three images were positive, the sample was confirmed ANA positive and the specific fluorescence patterns was identified by the laboratory technologist.

Anti-TPO antibodies were detected using the Kryptor immunoflourecent assay, BRAHMS (Hennigdorf, Germany), following the manufacturer’s manual. A concentration ≥ 60 kU/l was considered positive.

LA was detected by a simplified dilute Russell’s viper venom test (Siemens Healthcare Diagnostics Products GmbH, Marburg, Germany). The assay investigates the ratio of the coagulation time of patient plasma mixed with the LA1 screening reagent and the coagulation time after mixing plasma with the LA2 confirmatory reagent. If the ratio was > 1.2, LA was considered to be present.

aCL and β2GPI antibody of IgM and IgG isotypes were detected by a chemiluminescence 2-step immunoassay with magnetic particle separation (Bio-Flash, Werfen, Bruxelles, Belgium). A clinically relevant cut-off value for both antibodies was set to 35 kU/l.

### 2.4 Subgroups

The patients were divided into subgroups based on whether or not they had a low p-MBL level, the HLA-DRB1*03 phenotype and/or the HLA-DRB1*07 phenotype, respectively.

### 2.5 Statistical analysis

Data was collected from the RPL Center’s database (Data Protection Agency of The North Denmark Region, Approval Number 2018-5) and Stata ^®^ (MP 15.0 for Mac, revision 19, June 2017) was used for the statistical analyses. Categorical variables were compared using χ^2^-test, while when less than five observations were expected in a group, Fisher’s exact test was used. OR and 95% confidence intervals (CI) were calculated using univariate logistic regression. No attempt was made to adjust for potential confounding due to the sample size. A p-value < 0.05 was considered significant.

## 3 Results

In total, 644 RPL patients were included of whom 88.7% had three or more pregnancy losses. The majority had pregnancy losses after natural conception while 36.2% were supported with assisted reproductive technology (ART) treatment including *in-vitro* fertilization (IVF) and intra-cytoplasmatic sperm injection (ICSI).

The frequencies of the HLA-DRB1*03 and -DRB1*07 phenotypes in all RPL patients were 20.8.% and 19.6%, respectively, compared with 24.9% and 18.4%, respectively, in a Danish bone-marrow donor cohort (p=0.034; OR = 0.79, 95% CI 0.64-0.98 and p=0.532; OR 1.08, 95% CI 0.87-1.35 for comparisons between patients and controls with each of the two phenotypes) (22).

When comparing baseline characteristics between patients with low or normal p-MBL levels, the HLA-DRB1*03 and/or HLA-DRB1*07 phenotype, no clinically relevant or statistically significant differences were observed (**Table 1**).

**Table 1:** Baseline characteristics in all RPL patients and when grouping the patients according to plasma-MBL level, HLA-DRB1*03 or HLA-DRB1*07 phenotype, respectively.

	All RPL	Mannose-binding lectin plasma level		HLA-DRB1*03		HLA-DRB1*07	
		≤500	>500	Pos	Neg	Pos	Neg
	(N= 644)	(N= 237)	(N= 407)	(N= 134)	(N= 510)	(N= 126)	(N= 518)
**Age,** mean SD	33 5.2	33.1 5.0	33.0 5.0	32.7 5.3	33.1 5.1	33.1 5.0	33.0 5.2
**BMI,** median 25^th^-75^th^ percentile	25.0 22.3-29.0	24.5 22.0-28.5	25.3 22.5-29.0	26.0 22.0-29.0	24.9 22.5-28.5	25.1 23.0-28.5	24.9 22.0-20.0
**Pregnancy losses,** median 25^th^-75^th^ percentile	3 3-4	3 3-4	3 3-4	3 3-4	3 3-4	3 3-4	3 3-4
**sRPL,** N %	321 49.8%	120 49.4%	201 49.4%	63 47.0%	258 50.6%	69 54.8%	252 48.7

RPL, recurrent pregnancy loss; sRPL, secondary recurrent pregnancy loss.

The frequency of each autoantibody or autoantibody class did not vary between patients with low or normal p-MBL levels, and presence of the HLA-DRB1*03 or HLA-DRB1*07 phenotype, respectively. (**Table 2**). We observed non-significantly higher frequencies of ≥2 autoantibodies in patients with low p-MBL level compared to normal p-MBL level (p=0.14) and in patients with minimum one HLA-DRB1*03 allele compared with patients with no HLA-DRB1*03 allele (p=0.11). Also, a tendency was found for a lower frequency of APL antibodies among patients with minimum one HLA-DRB1*07 allele compared to patients with no HLA-DRB1*07 allele (p=0.08) (**Table 2**).

**Table 2:** The frequencies of antibody positivity in all RPL patients and when grouping the patients according to plasma-MBL level, HLA-DRB1*03, or HLA-DRB1*07 phenotype, respectively.

	All RPL	Mannose-binding lectin plasma level			HLA-DRB1*03			HLA-DRB1*07		
	Total	≤500 µg/l	>500 µg/l		Pos	Neg		Pos	Neg	
**AB**	(N= 644) N, %	(N= 237) N, %	(N= 407) N, %	**P**	(N= 134) N, %	(N= 510) N, %	**P**	(N= 126) N, %	(N=518) N, %	**P**
**ANA**	82 12.7%	35 14.8%	47 11.6%	0.24	22 16.4%	60 11.8%	0.15	18 14.3%	64 12.4%	0.56
**TPO**	86 13.4%	36 15.2%	50 12.3%	0.30	18 13.4%	68 13.3%	0.98	14 11.1%	72 13.9%	0.41
**APL**	50 7.7%	19 8.0%	31 7.6%	0.86	12 9.0%	38 7.5%	0.56	5 4.0%	45 8.7%	0.08
**1 AB**	148 23.0%	55 23.2%	93 22.9%	0.76	28 20.9%	120 23.5%	0.66	30 23.8%	118 22.8%	0.84
**≥1 AB**	183 28.4%	72 30.4%	111 27.3%	0.39	39 29.1%	144 28.2%	0.84	36 28.6%	147 28.3%	0.97
**≥2 AB**	35 5.4%	17 7.2%	18 4.4%	0.14	11 8.2%	24 4.7%	0.11	6 4.8%	29 5.6%	0.71

AB, antibody; Pos, positive; Neg, Negative; ANA, antinuclear antibody; TPO, thyroid-peroxidase; APL, antiphospholipid.

Among patients who who were positive for one or more autoantibodies and had low p-MBL level, the frequency of carrying an HLA-DRB1'03 allele was higher than among those with one or more autoantibodies and normal p-MBL level (OR = 2.4, 95% CI 1.2-5.0; p = 0.01) (**Table 3**). All other pairwise comparisons of HLA-DRB1 phenotype frequencies between subsets of patients divided according to presence/absence of autoantibodies and p-MBL levels showed no significant differences (**Table 3**). Moreover, when examining the HLA-DRB1*03/X genotypes, no differences between patients with low or normal p-MBL in women without autoantibodies were observed. However, among patients positive for ≥1 autoantibody, notable differences in HLA-DRB1*03/X genotypes were seen between patients with low or normal p-MBL levels; however, none of these differences were significant. (**Table 4**).

**Table 3:** The frequency of carrying at least one of the HLA-DRB1 alleles in patients grouped according to presence or absence of at least one of the investigated autoantibody classes and low or normal plasma MBL levels.

HLA-DRB1 phenotype	0 AB			≥1 AB		
	MBL ≤500 µg/l N_total_=165 N (%)	MBL >500 µg/l N_total_=296 N (%)	P	MBL ≤500 µg/l N_total_=72 N (%)	MBL >500 µg/l N_total_=111 N (%)	P
**01**	31 (18.8)	66 (22.3)	0.38	13 (18.1)	23 (20.7)	0.66
**03**	35 (21.2)	60 (20.3)	0.81	22 (30.6)	17 (15.3)	**0.01**
**04**	53 (32.1)	92 (31.1)	0.82	24 (33.3)	43 (38.7)	0.46
**07**	31 (18.8)	59 (19.9)	0.77	12 (16.7)	24 (21.6)	0.41
**08**	14 (8.5)	17 (5.7)	0.26	8 (11.1)	9 (8.1)	0.49
**09**	5 (3.0)	10 (3.4)	1.0	1 (1.4)	4 (3.6)	0.65
**10**	3 (1.8)	4 (1.4)	0.71	1 (1.4)	2 (1.0)	1.0
**11/12**	32 (19.4)	48 (16.2)	0.39	17 (23.6)	23 (20.7)	0.54
**13**	49 (29.7)	77 (26.0)	0.40	15 (20.8)	26 (23.4)	0.68
**14**	7 (4.2)	22 (7.4)	0.18	7 (9.7)	6 (5.4)	0.27
**15**	46 (27.9)	88 (29.7)	0.68	14 (19.4)	30 (27.3)	0.24
**16**	2 (1.2)	5 (1.7)	1.0	3 (4.2)	3 (2.7)	0.68

AB, antibody; MBL, mannose binding lectin. The bold values denote statistically significant P-values.

**Table 4:** The frequency of carrying at least one of the HLA-DRB1*03/X genotypes in patients grouped according to presence or absence of at least one of the investigated autoantibody classes and low or normal plasma MBL levels.

HLA-DRB1 genotype	0 AB			≥1 AB		
	MBL ≤500 µg/l N_total_ = 35 N (%)	MBL >500 µg/l N_total_ = 60 N (%)	P^a^	MBL ≤500 µg/l N_total_ = 22 N (%)	MBL >500 µg/l N_total_ = 17 N (%)	P^b^
**01/03**	5 (14.3)	8 (13.3)	1.0	4 (18.2)	1 (5.9)	0.36
**03/03**	3 (8.6)	4 (6.7)	1.0	1 (4.5)	1 (5.9)	1.0
**03/04**	7 (20.0)	12 (20.0)	1.0	1 (4.5)	4 (23.5)	0.15
**03/07**	5 (14.3)	8 (13.3)	1.0	3 (13.6)	0 (0.0)	0.24
**03/08**	1 (2.9)	0 (0.0)	0.37	2 (9.1)	3 (17.6)	0.64
**03/09**	1 (2.9)	3 (5.0)	1.0	0 (0.0)	0 (0.0)	–
**03/10**	0 (0.0)	0 (0.0)	–	0 (0.0)	0 (0.0)	–
**03/11 or 03/12**	3 (8.6)	5 (8.3)	1.0	2 (9.1)	2 (11.8)	1.0
**03/13**	5 (14.3)	9 (15.0)	1.0	2 (9.1)	1 (5.9)	1.0
**03/14**	0 (0.0)	2 (3.3)	0.53	2 (9.1)	1 (5.9)	1.0
**03/15**	4 (11.4)*	8 (13.3)*	1.0	5 (22.7)*	4 (23.5)*	1.0
**03/16**	1 (2.9)	1 (1.7)	1.0	0 (0.0)	0 (0.0)	–

AB, antibody; MBL, mannose-binding lectin ^a,b^ Comparing patients with low or normal p-MBL; *, comparing patients without autoantibodies with genotype HLA-DRB1*03/15 versus patients with autoantibodies without the genotype, p = 0.13.

ANAs were found with increased prevalence in patients with the combination of HLA-DRB1*03 and low p-MBL level compared with those without this combination (OR 2.0; 95% CI 1.0 - 3.9; p <0.05). The presence of the all other autoantibodies was also increased in patients with HLA-DRB1*03 and low p-MBL (ORs between 1.5 and 1.7) but these associations were not statistically significant (**Table 5**).

**Table 5:** The frequency of each of the three autoantibody classes in patients with or without the HLA-DRB1*03/low plasma MBL combination.

HLA-DRB1 phenotype and p-MBL level combination								
AB	DRB1*03 pos N_total_ =134		DRB1*03 pos and MBL ≤500 µg/l N_total_=57		DRB1*03 neg and/or MBL >500 µg/l N_total_=587		OR (95% CI)	p
	B	%	N	%	N	%		
**ANA**	22	16.4	12	21.1	70	11.9	2.0 (1.0-3.9)	**0.05**
**TPO**	18	13.4	11	19.3	75	12.8	1.7 (0.8-3.2)	0.17
**APL**	12	9.0	6	10.5	44	7.5	1.5 (0.6-3.6)	0.41

AB, antibody; OR, odds ratio; CI, confidence interval; ANA: antinuclear antibody; TPO, thyroid-peroxidase; APL, antiphospholipid; MBL, mannose-binding lectin; ^a^Two patients had missing ANA measurement and one had missing TPO measurement. The bold values denote statistically significant P-values.

## 4 Discussion

In this study, we found that the combination of carriage of HLA-DRB1*03 and p-MBL levels ≤500 µg/l is associated with presence of autoantibodies especially ANAs in patients with RPL.

In previous large case-control studies, we have reported that HLA-DRB1*03 and HLA-DRB1*07 are conferring susceptibility to RPL (21, 22). Only the studies published by the groups of Christiansen et al. found the HLA-DRB1*03 frequency significantly increased in women with RPL compared with controls (21, 25). In a meta-analysis of 18 case-control studies investigating HLA-DR phenotype frequencies in RPL patients and controls, exclusion of patients positive for autoantibodies was done in 5 of the 7 studies finding HLA-DRB1*03 less frequent in RPL patients than in controls. In contrast, exclusion of autoantibody-positive patients was not done in any of the studies finding HLA-DRB1*03 increased in RPL patients (26). Thus, such selection bias may have distorted the observed HLA-DR distribution in the RPL cohort, and it is likely that the meta-analysis would have shown HLA-DRB1*03 significantly associated with RPL if a sensitivity analysis had been done excluding the 5 studies where autoantibody positive RPL patients were excluded.

The overall frequency of the HLA-DRB1*03 phenotype in RPL women in this study was lower than the frequency in a large previously published cohort of Danish bone marrow donors (22). We have previously reported an increasing population incidence of RPL (27) and a declining frequency of HLA-DRB1*03 among RPL patients (22). The declining frequency of the allele in RPL patients was confirmed in the present study. This pattern of temporal weakened effect of some susceptibility HLA alleles in diseases has also been described for type I diabetes mellitus (28, 29). It has been speculated whether the declining frequency of susceptibility HLA alleles in patients together with the increasing population incidence of type I diabetes are due to an increasing proportion of diabetes type I cases being caused by environmental and lifestyle changes (28, 29). In analogy with the studies in diabetes type I, we speculate whether HLA susceptibility alleles (and especially HLA-DRB1*03) with time play a declining role whereas environmental or lifestyle factors could play an increasing role in the RPL pathogenesis. However, a deeper insight into the complex and probably multifactorial pathophysiology causing RPL is needed before we can make a realistic estimate of the contribution of immunogenetic, environmental, and lifestyle factors to RPL.

More simple explanations for the failure to confirm the association previously found between HLA-DRB1*03 and RPL (21) are possible. A small statistical power may explain why the present study did not detect the association, however, now two large, independent studies of non-overlapping cohorts including 1078 (22) and 663 (this study) RPL patients, respectively, have not been able to confirm the association. Therefore, the lack of statistical power is a less likely explanation. Another possible explanation is a changed referral practice. The vast majority of the RPL patients in the prior study finding an association (21) had conceived spontaneously and had a history of predominantly clinical miscarriages confirmed by ultrasound scans whereas approximately 1/3 of RPL patients in this study had conceived by ART (IVF or ICSI) and an increased proportion of their pregnancy losses were biochemical pregnancies due to the change in the definition of RPL by European Society of Human Reproduction and Embryology’s Guideline group in the meantime between the two studies. The immunological component in the pathogenesis of the losses in our current RPL patients may have been weakened by such changes in patient characteristics.

We have in repeated studies in different populations of RPL patients and controls found that low levels of p-MBL are associated with increased risk of RPL with ORs between 1.46 and 1.79 (23, 24). Moreover, the most recent study high p-MBL levels (≥3000µg/l) seemed to protect against RPL (23). In the present study, most blood samples were taken prior to pregnancy whereas 10.2% were taken in the first trimester. A previous study found that only a minor elevation of p-MBL levels could be detected from before pregnancy to the first trimester (30). Since the huge majority of our patients’ pregnancy losses happen in the first trimester, we believe that the measurements made before or in early pregnancy reflect the p-MBL levels in the high-risk period for pregnancy loss and therefore, the MBL measurement from both timepoints are relevant. Although being considered an acute-phase protein (31), p-MBL only seems to increase in response to infections in individuals with MBL-2 genotypes predisposing to high p-MBL levels (31) so low p-MBL levels <500 µg/l are considered to be stable also during infection/inflammation.

In patients with unexplained RPL, various autoantibodies can be detected with higher prevalence than in controls, and they are thought to have a negative prognostic impact. In the European Society of Human Reproduction and Embryology’s Guideline for RPL (12), it is recommended to screen RPL patients for LA, anti-TPO antibodies, ANAs, and APL antibodies – and among APL antibodies especially aCL antibodies since plenty of studies have documented the association between aCL antibodies and RPL whereas only few studies have documented the association between β2GPI antibodies and RPL (4). Although these autoantibodies seem to affect the prognosis in RPL patients negatively, it is unclear whether they act directly to the fetus and trophoblast and cause miscarriage, whether they are markers for an overall dysregulated immune response towards pregnancy, or whether they are confounding factors associated with more basic risk factors such as genetic factors of importance for immune regulation.

As HLA-DRB1 polymorphism and MBL deficiency are both associated with autoimmunity and with RPL separately, the natural question arises whether autoantibody development is an intermediate or causal factor between the immunogenetic profile and occurrence of RPL. We tried to answer this question in a former study: In a case-control study of Danish and Czech patients with RPL, Christiansen et al. (32) found a significant difference between the prevalence of patients being HLA-DRB1*03 positive among ANA positive and ANA negative patients (55% vs 28%, p <0.05) and a significant difference in the prevalence of the allele between aCL antibody-positive patients and the background population (35% vs 21%, p < 0.05). It was concluded that HLA-DRB1*03 is a susceptibility gene for production of the two autoantibodies in Caucasian RPL women.

We performed an updated study on the association between relevant immunogenetic biomarkers and autoantibody production in a larger population of RPL patients and tested more associations than previously: between 3 genetically determined biomarkers - two HLA class II gene variants and MBL deficiency against 3 classes of autoantibodies. The study illustrates that several genetic variants interact in the pathogenesis of autoimmunity in RPL patients. Essentially, we confirmed the previously reported statistically significant association between ANA and HLA-DRB1*03 but now only in the subset of patients with MBL deficiency. This disparity may reflect the previously mentioned weakened effect of HLA class II susceptibility alleles over time seen in some autoimmune disorders.

Although we have recently reported that HLA-DRB1*07 is significantly associated with RPL (22), it is evident that this allele is not associated with production of the tested autoantibodies (**Table 2**). The pathophysiological mechanisms causing RPL in HLA-DRB1*07 positive patients seem not to involve autoantibody production but may instead be mediated by HLA-DRB1*07 restricted T-cells directed against male-specific minor histocompatibility antigens on the fetus or trophoblast (33).

No study has so far clarified which mechanisms underly the association between RPL and low plasma MBL. There could be several mechanisms. One mechanism may be related to MBL’s ability to bind to and promote clearance of necrotic and apoptotic cells and debris probably through opsonizing these cells and particles and promoting phagocytosis by mainly macrophages (34). If apoptotic trophoblast cells or debris shed from the placenta are not rapidly cleared by this mechanism, they may instead be phagocytosed by endometrial endothelial cells, which may result in increased release of proinflammatory cytokines (35). The increased proinflammatory response may injure the endothelial cells in placenta and consequently cause vascular clotting and trophoblast/fetal ischemia. Thus, pregnant women with low p-MBL may be at a higher risk for harmful proinflammatory responses at the feto-maternal interface due to delayed clearance of apoptotic trophoblast cells/debris.

MBL may also, in theory, affect antibody/autoantibody production through more direct ways since it can bind to and influence B lymphocytes (36) and since mice studies have found increased proliferation and IgG production in MBL null mice compared to wild type in response to Group B Streptococcus vaccination (37).

Another mechanism may be related to the results from previous reports suggesting that blocking asymmetric IgG antibodies possessing mannose-rich oligosaccharide residues in the Fab residue may play a role in RPL since the levels of such antibodies increase during normal pregnancy but not in pregnancies of women with RPL (38). MBL may bind to the mannose-rich residues of the asymmetrical antibodies enhancing their blocking effect (also on autoantibody production) whereas in women with low p-MBL, the blocking effect of asymmetrical antibodies may be reduced.

More experimental studies are needed to test these hypotheses. In our Center, we are currently performing studies in order to rule out whether the clearance rate of fetal and trophoblast antigens after a delivery is depending on the p-MBL level.

The finding in this study that an interaction of HLA-DRB1*03 and MBL is associated with the presence of autoantibodies in RPL is new. Only a weak association was found between HLA-DRB1*03 and ANA, and no overall association between this allele and the other investigated autoantibodies. These findings may be surprising since previous studies have found HLA-DRB1*03 being associated with the presence of a series of autoimmune diseases. However, almost all previous studies have focused on patients with clinical autoimmune disease (39–42) where the autoimmune reactions causing their clinical symptoms are more pronounced than in otherwise healthy RPL patients who typically is less or not at all affected in terms of clinical symptoms when not pregnant potentially because of lower autoantibody titers. This may explain the absent/weak association to solitary HLA-DRB1*03 found this study (**Table 2**). The finding that no other HLA-DRB1 allele than HLA-DRB1*03 seems to interact with low MBL is a new but negative finding, however, it does contribute to our knowledge about the pathogenesis of RPL. New independent studies are, however, needed to confirm these results.

It is recommended that a panel of relevant immunogenetic variants are investigated in patients with RPL and that models are developed for the seemingly complex interaction between the variants and the risk of RPL.

## Data availability statement

The original contributions presented in the study are included in the article/supplementary material. Further inquiries can be directed to the corresponding authors.

## Ethics statement

Ethical review and approval was not required for the study on human participants in accordance with the local legislation and institutional requirements. The patients/participants provided their written informed consent to participate in this study.

## Author contributions

CN-P collected data and performed statistical analyses. CN-P and OC analyzed the results and wrote the article. RS was responsible for the HLA genotyping and p-MBL measurements. UK critically reviewed the manuscript and the statistics. All authors contributed to the article and approved the submitted version.
